# Ameliorative Hematological and Histomorphological Effects of Dietary *Trigonella foenum-graecum* Seeds in Common Carp (*Cyprinus carpio*) Exposed to Copper Oxide Nanoparticles

**DOI:** 10.3390/ijerph192013462

**Published:** 2022-10-18

**Authors:** Aasma Noureen, Giuseppe De Marco, Nagina Rehman, Farhat Jabeen, Tiziana Cappello

**Affiliations:** 1 Department of Zoology, Government College Women University Faisalabad, Faisalabad 38000, Pakistan; 2 Department of Biology, Virtual University of Pakistan, Faisalabad 38000, Pakistan; 3 Department of Chemical, Biological, Pharmaceutical and Environmental Sciences, University of Messina, 98166 Messina, Italy; 4 Department of Zoology, Government College University Faisalabad, Faisalabad 38000, Pakistan

**Keywords:** nanoparticle toxicity, freshwater fish, histomorphology, blood parameters, oxidative stress, fenugreek seed extract

## Abstract

Different types of metal oxide nanoparticles (NPs) are being used for wastewater treatment worldwide but concerns have been raised regarding their potential toxicities, especially toward non-targeted aquatic organisms including fishes. Therefore, the present study aimed to evaluate the toxicity of copper oxide (CuO) NPs (1.5 mg/L; positive control group) in a total of 130 common carp (*Cyprinus carpio*), as well as the potential ameliorative effects of fenugreek (*Trigonella foenum-graecum*) seed extracts (100 mg/L as G-1 group, 125 mg/L as G-2 group, and 150 mg/L as G-3 group) administered to fish for 28 days. Significant changes were observed in the morphometric parameters: the body weight and length of the CuO-NP-treated fish respectively decreased from 45.28 ± 0.34 g and 14.40 ± 0.56 cm at day one to 43.75 ± 0.41 g and 13.57 ± 0.67 cm at day 28. Conversely, fish treated with *T. foenum-graecum* seed extract showed significant improvements in body weight and length. After exposure to CuO NPs, a significant accumulation of Cu was recorded in the gills, livers, and kidneys (1.18 ± 0.006 µg/kg ww, 1.38 ± 0.006 µg/kg ww, and 0.05 ± 0.006 µg/kg ww, respectively) of the exposed common carp, and significant alterations in fish hematological parameters and oxidative stress biomarkers (lipid peroxidation (LPO), glutathione (GSH), and catalase (CAT)) were also observed. However, supplementing diets with fenugreek extracts modulated the blood parameters and the oxidative stress enzymes. Similarly, histological observations revealed that sub-lethal exposure to CuO NPs caused severe histomorphological changes in fish gills (i.e., degenerative epithelium, fused lamellae, necrotic lamellae, necrosis of primary lamellae, complete degeneration, and complete lamellar fusion), liver (i.e., degenerative hepatocytes, vacuolization, damaged central vein, dilated sinusoid, vacuolated degeneration, and complete degeneration), and kidney (i.e., necrosis and tubular degeneration, abnormal glomerulus, swollen tubules, and complete degeneration), while the treatment with the fenugreek extract significantly decreased tissue damage in a dose-dependent manner by lowering the accumulation of Cu in the selected fish tissues. Overall, this work demonstrated the ameliorative effects of dietary supplementation with *T. foenum-graecum* seed extract against the toxicity of NPs in aquatic organisms. The findings of this study therefore provided evidence of the promising nutraceutical value of fenugreek and enhanced its applicative potential in the sector of fish aquaculture, as it was shown to improve the growth performance and wellness of organisms.

## 1. Introduction

Nanoparticles (NPs) are well defined according to their unique properties such as a large surface-area-to-volume ratio and nanoscale size, which make them very reactive and applicable in various scientific fields [1,2]. Metal oxide NPs are considered to be one of the most abundantly used subsets of nanomaterials; depending upon their different forms and compounds, these NPs have been increasingly employed in a vast range of applications [2,3]. In particular, copper- and copper oxide-based NPs (Cu and CuO NPs) are broadly used in several industrial sectors including construction [4], electronics [5], in organic synthesis as catalysts [6], as antimicrobial agents [7], in food preservation and in the agriculture sector [8,9], for biomedical applications in novel drug-delivery systems [10], and in paint and water management [11,12]. In light of the rapid development of nanotechnology globally and the widespread use of CuO NPs in various fields, concerns have been raised regarding their potential ecotoxicity to humans and aquatic organisms, including fish [13,14,15,16].

As a matter of fact, although the beneficial effects of nanotechnologies are undeniable, the excessive usage of NPs in day-to-day life and their subsequent release into aquatic ecosystems pose major health risks for the environment [17], including freshwater and marine habitats [18,19]. The major reason behind the hazard attributable to metal NPs is related to their dispersion and tendency to bioaccumulate into biological tissues. Indeed, due to their nanoscale dimensions, NPs may easily pass through membranes and biological barriers and therefore interfere with the normal functions of various organ systems [20,21]. As a result, exposure to metal NPs causes damage at the cellular and sub-cellular level, triggering a variety of cell/tissue responses in aquatic biota [3,21,22,23,24,25,26]. The major adverse effects provoked by CuO NPs in fish were summarized in a recent comprehensive review on the toxicological impacts of these NPs in fish [16] that reported that CuO NPs caused inhibition of Na^+^/K^+^-ATPase activity, a decline in plasma Na^+^ and Cl^−^, arterial O_2_ tension, cell swelling, thickening and curling of the gill lamellae, cytolysis, necrosis, pyknosis, and even fibrosis. Moreover, an increased mortality rate and reduced reproductive success were also documented in guppy fish after exposure to acute and chronic exposure of CuO NPs [27].

Among the various therapeutic strategies being used to treat nanotoxicities, an increasing trend in the use of herbal medicines is being seen because they have been used for thousands of years and almost 80% of world’s population relies on them for primary health care. Among the various medicinal plants (including garlic, ginger, green tea, cinnamon, and moringa) that have been used for many protective roles, fenugreek (*Trigonella foenum-graecum*) is also extensively used in countries of the Orient as a spice due to its aroma and nutritional value [28]. Moreover, numerous studies demonstrated the antioxidant potential of *T. foenum-graecum,* which greatly increased scavenger enzymatic activities and reduced the production of reactive oxygen species (ROS) as observed in diabetic rats [29,30]. Additionally, in vitro studies documented the potential of fenugreek aqueous extracts in reducing fat deposition in rats [31], as well as the free radical scavenging properties of *T. foenum-graecum* oil and its potent antioxidant characteristics [32]. Among the positive effects of dietary supplementation of fenugreek in fish, its ability to promote growth performance of gilthead seabreams and augment various blood parameters, including the number of erythrocytes, was reported [33]. The cytoprotective properties of fenugreek with mitigation of tissue alteration provoked by cadmium exposure were also observed in Nile tilapia [34]. Moreover, the beneficial protective effects of *T. foenum-graecum* against many toxicants such as carbon tetrachloride [35], cadmium [36], aluminum [37], and deltamethrin [38] have been also reported in a variety of animals, especially mammals. However, to our knowledge, no data are available on the potential curative effects that fenugreek may have against NP-induced toxicities and there is still scarce information about its possible successful use in fish diets for a sustainable aquaculture [33,34].

Common carp (*Cyprinus carpio*) is a commercially relevant freshwater fish species that is mainly distributed in Asia and Europe. It is considered to be a very important aquaculture species worldwide due to its easy rearing, low-cost production, and elevated muscle contents with a high nutritional value [39]. In addition to their economic importance, common carp are also considered to be a valid freshwater animal model and are one of the most used freshwater fish in ecological, developmental, and ecotoxicological studies [24,25,26,40]. Its peculiarities as a model fish species for toxicity studies are related to its high tolerance toward toxicants and its capacity to adapt to a variety of habitats. Therefore, while taking into account the urgent need to unravel the impact of NPs on aquatic organisms, the present study was designed to assess the toxicity of CuO NPs in *C. carpio* in terms of the hematological profile; histology of fish gills, livers, and kidneys; and antioxidant defense systems; as well as to elucidate the potential ameliorative effects of fenugreek (*T. foenum-graecum*) seed extracts on the health status of common carp exposed to CuO NPs.

## 2. Materials and Methods

### 2.1. Procurement and Acclimatization of Fish

Common carp (*Cyprinus carpio*; 40–45 g body weight) were procured from the Fish Seed Hatchery on Satiana Road in Faisalabad, Pakistan. The fish were then transported in plastic containers with continuous aeration to the laboratory of the Department of Zoology, Government College University Faisalabad (GCUF), Pakistan, and were acclimatized in a 100 L tank for two weeks prior to the experiment. The water temperature was maintained up to 25 °C and the dissolved oxygen and pH were kept at 6.8–7.4 mg/L and 6.9–7.2, respectively. The concentration of ammonia (NH_3_), total hardness, and total dissolved solids were 0.4–0.6 ppm, 47–52 ppm, and 6.8–7.5 ppt, respectively. During the acclimatization period, the fish were kept under a normal photoperiod (12 h light:12 h dark) and fed twice daily with commercial fish feed (Super Nova, SKU103892996_PK-1250174609). The water was regularly changed daily and dead fish were removed.

### 2.2. Preparation of CuO NP Suspensions

The CuO NPs were acquired as a powder from COMETOX and were characterized by a particle size of 12 nm, a molecular weight of 79.55, and a purity of 99.5% according to data provided by the manufacturer. The characterization of the CuO NPs, which was reported in our previous papers [21,22], was performed by using a field emission scanning electron microscope (FESEM, FEI FESEM, FEI, Quanta 200 Company, Thermo Fisher Scientific, Waltham, MA, USA) that revealed a tendency of the CuO NPs to form aggregates with a mean size of 80 nm [21]. Moreover, additional data were determined by using dynamic light scattering (DLS), such as the polydispersity index (0.25), the hydrodynamic radius (140 nm), and the zeta potential (−6.5 ± 0.5 mV) of CuO NPs [22]. For the preparation of the exposure medium, the required amounts of CuO NPs were weighed by using a laboratory weight balance (Model HC series) and placed into polypropylene tubes (Pyrex) and mixed with ultra-pure water (Millipore, 18.2 Mcm resistance, Thermo Fisher Scientific, Waltham, MA, USA) without any solvent. The suspension was homogenized by using a vortex (Vortex Genie-2T, Thermo Fisher Scientific, Waltham, MA, USA) for 3 h at 2000 rpm to obtain the maximum dispersion and then was ultrasonicated (Jp-031) for 1 h prior to each dosing.

### 2.3. Identification and Preparation of the Fenugreek Extract

Dried seeds of *T. foenum-graecum* were procured from authenticated stores and identified taxonomically at the Department of Botany, GCUF, Pakistan. Seeds were ground, sieved, and stored in an air-tight jar for further analysis, including extract preparation. The plant extract was prepared by using the standard method of Khan et al. [41] with a few modifications. In brief, 500 g of powdered plant material was collected and mixed with methanol in a ratio of 1:1 and kept at room temperature for one week. After that, it was mixed and filtered by using Whatman filter paper No. 1. The extract was then dried, weighed, and stored for further analysis.

The chemical/proximate composition of the selected plant was determined as described by the Association of Official Analytical Chemists (AOAC) [42]. Additionally, for exploration of the secondary metabolites, standard protocols were applied for the total soluble phenolics [43], tannins [44], alkaloids, saponins [43], and flavonoids [45].

### 2.4. Evaluation of 96 h LC50 of Fenugreek for C. carpio

To evaluate the 96 h LC50 (or median lethal concentration) value of *T.* *foenum-graecum* for *C. carpio*, the fish were first treated with fenugreek extracts used as antioxidants. For this purpose, the fish were exposed in triplicate to different doses of the plant extracts of 125, 250, 500, 1000, or 2000 mg/L concentrations of *T.* *foenum-graecum* separately. The experiment was carried out in glass aquaria of with a 40 L water capacity and with the same physico-chemical parameters applied during the acclimatization period. The test water was not changed during the 96 h time period and the exposed fish were not fed. The fish were removed after death on a daily basis in the early morning and late evening. The 96 h LC50 was calculated by using Probit Analysis (Minitab® 17.1.0 software, © 2013 Minitab Inc., State College, PA, USA).

### 2.5. Experimental Design

A total of 130 fish of equal weight were distributed into four treated groups, including one positive control group (C-positive) treated with a sub-acute selected dose of CuO NPs at 1.5 mg/L and three other treated groups (G-1, G-2, and G-3) reared with three different doses of *T. foenum-graecum* (100, 125, or 150 mg/L, respectively) along with the selected dose of CuO NPs through waterborne exposure, in addition to one negative control group (C-negative or control) with fish exposed to no treatment with free access to food. A dose of 1.5 mg/L of the CuO NPs was selected in accordance with our previous studies by Noureen et al. [25,26], in which a pilot study was conducted in order to establish the 96 h LC50 and sub-acute toxicity of the CuO NPs in fish based on the sub-lethal doses for the 96 h LC50 of Cu NPs in common carp. The experimental exposures were conducted for 28 days in triplicates. The water’s physico-chemical parameters such as temperature, pH, total dissolved solids, and oxygen were determined by using a multiparameter apparatus (HI 9828, Hanna Instruments, Keison Co., Chelmsford, UK), while total ammonia and water hardness were assessed by using titration methods [46].

The fish were anesthetized with 75 mg/L of clove oil in bucket for 4 min [47,48]. Blood samples were drawn from the caudal vein using a sterile syringe and then transferred in EDTA microtubes [49,50,51] for hematological analysis. Tissues of gills, livers, and kidneys were collected, properly processed, and then stored at −20 °C until the analyses for metal accumulation and oxidative stress enzymes were conducted. Moreover, small pieces of each dissected tissue were taken for histological analysis and immediately fixed in a Bouin solution.

The in vivo experiments with fish were carried out with approval from the Animal Ethics Committee, GCUF, Pakistan. The experimental fish received proper care and husbandry in compliance with the Animal Ethics Committee’s guidelines and the minimum possible number of fish was used in this experiment.

### 2.6. Biochemical Parameters

#### 2.6.1. Tissue Metal Analysis

Samples weighing 1 g for each tissue (gills, liver, and kidney) were transferred to a digestion flask (Pyrex) and mixed with 10 mL of concentrated nitric acid (HNO_3_) and 2 mL of perchloric acid (HClO_4_) and then heated on a hot plate (6796-620D) using a fuming hood at 100 °C until the color disappeared. The samples were then cooled and diluted with 50 mL of deionized water and filtered using Whatman filter paper No. 1 [52]. The absorbance was detected by using an atomic absorption spectrometer (AI 1200, Aurora Instruments LTD, Thermo Fisher Scientific, Waltham, MA, USA) and is expressed as µg/kg wet weight (ww).

#### 2.6.2. Hematological Analysis

Assessments of red blood cells (RBC), white blood cells (WBC), hemoglobin (HGB), platelets (PLT), lymphocytes (LYM), mean cell volume (MCV), mean cell hemoglobin (MCH), mean cell hemoglobin concentration (MCHC) and hematocrit (HCT) were conducted by using a hematology automated analyzer (Beckman Coulter UniCel DxH, Thermo Fisher Scientific, Waltham, MA, USA) according to manufacturer’s guidelines.

#### 2.6.3. Histological Analysis

The tissues fixed in Bouin solution were further processed using an ascending grade of alcohol; i.e., 70–100%, followed by a xylene solution. After that, the samples were fixed in a paraffin wax and then slides with an approximately 5 µm thickness were cut using a microtome (Histo-line, MR2258, Thermo Fisher Scientific, Waltham, MA, USA). The tissue slides were deparaffinized and rehydrated using a descending grade of alcohol; i.e., from 100% to 30%, and then stained using hematoxylin and eosin (H&E staining) according to protocols described previously [53,54,55]. Histological observations were carried out on five fields of one section per sample at 400× magnification using a light microscope (MEIJI, Model: MT4300H, Saitama, Japan), equipped with a Canon digital camera (EOS 1300D) for acquisition of photographs.

#### 2.6.4. Analysis of Oxidative Stress Enzymes

The freshly excised fish liver and gills were washed with a buffer and soaked in 10% homogenate using 0.1 M of phosphate buffer (pH 7.5) using a Potter-Elvejham homogenizer (Thermo Fisher Scientific, Waltham, MA, USA). The homogenates were then centrifuged (Z32-HK) at 10,000 rpm for 10 min. The supernatants were collected and kept at −20 °C until the analysis of oxidative stress biomarkers [52] including lipid peroxidation (LPO) [56], glutathione (GSH) [57], and catalase (CAT) [58] by applying their respective protocols. Specifically, to estimate LPO, a measurement of malondialdehyde (MDA; nmol/mg protein) was performed by applying the thiobarbituric acid reactive (TBARS) method as described in detail elsewhere [59,60]. The reduced GSH was estimated using the colorimetric Ellman’s method [61], treating the tissue homogenate with trichloroacetic acid (TCA) 10%, and then adding 0.2 M Tris EDTA buffer in order to prevent GSH oxidation. In addition, the enzymatic activity of CAT (μmol/min/mg protein) was evaluated following the dismutation of H_2_O_2_ at 240 nm for 90 s using a colorimetric technique [59,60].

### 2.7. Statistical Analysis

Data were expressed as means ± standard error (SE; n = 10) and analyzed using Minitab17 software; we applied one-way ANOVA and the Tukey’s post hoc test for the comparative analyses. Data were considered statistically significant at *p* < 0.05.

## 3. Results

### 3.1. LC50 of T. foenum-graecum

**Figure 1:**
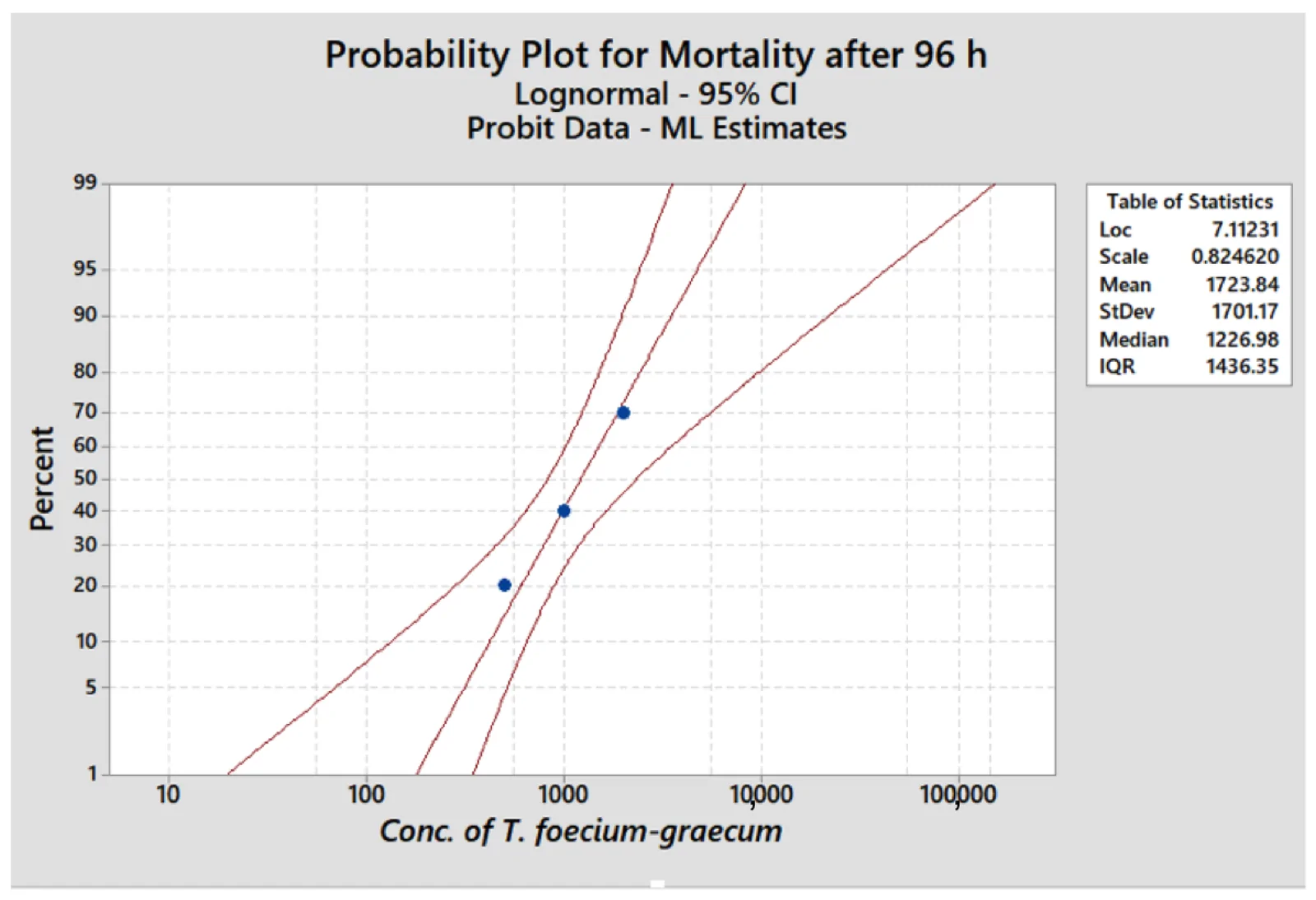
The mortality of *C. carpio* expressed as percent at different concentrations of *T. foenum-graecum* during the 96 h acute toxicity test.

The 96 h LC50 value of the fenugreek for *C. carpio* was 1226.98 ± 259.89 mg/L with a 95% confidence interval of 832.29 to 2354.72 mg/L, which showed the toxicity of the *T. foenum-graecum* extract to the common carp at a higher concentration (Figure 1).

### 3.2. Phytochemical and Proximate Composition of T. foenum-graecum

Phytochemical analysis of the seeds of fenugreek (*T. foenum-graecum*) revealed that the percentages of tannins (325.5 ± 9.10 mg/100 g of fenugreek) and alkaloids (9.52 ± 1.01 mg/100 g of fenugreek) were significantly (*p* < 0.05) higher compared to those of total phenolics (0.42 ± 0.01 mg/100 g of fenugreek), saponins (0.07 ± 0.02 mg/100 g of fenugreek) and flavonoids (0.64 ± 0.03 mg/100 g of fenugreek) in the following order: tannins > alkaloids > flavonoids > total phenolics > saponins.

Similarly, with regard to the proximate composition of the fenugreek, the concentrations expressed as percentage of carbohydrates (56.75 ± 4.01%) and oil contents (25.68 ± 1.81%) were significantly (*p* < 0.05) higher than those of fiber (14.56 ± 1.2%), proteins (12.24 ± 1.51%), moisture (9.22 ± 1.01%), and ash (5.35 ± 1.02%) in the following order: carbohydrates > oil contents > fiber > proteins > moisture > ash.

### 3.3. Effects on Fish Body Weight and Length

The 28-day exposure to the selected dose of CuO NPs caused a significant (*p* < 0.05) reduction in the body weight and length of the common carp. However, administration of fenugreek (*T. foenum-graecum*) induced a significant (*p* < 0.05) dose-dependent amelioration in the fish in terms of weight gain and increased body length in comparison with the CuO-NP-treated group (Table 1).

### 3.4. Bioaccumulation of Cu in Fish Tissues

The 28-day exposure to the selected dose of CuO NPs in common carp induced a significant (*p* < 0.05) accumulation of Cu in the gills, livers, and kidneys in comparison with fish from the non-treated group (C-negative). However, the treatments with different concentrations of *T. foenum-graecum* extract induced significant (*p* < 0.05) dose-dependent protective effects by reducing the accumulation level of Cu in the gills, livers, and kidneys of *C*. *carpio* (Table 2).

### 3.5. Hematological Profile

The hematological profile of the common carp with different parameters including Hb, Hct, RBC, WBC, MCV, MCH, MCHC, and platelet count is reported in Table 3, which shows the normal hematological values of fish from the negative control group together with those recorded in fish from the positive control and fenugreek-treated groups. The data revealed that the exposure to the CuO NPs provoked a significant (*p* < 0.05) depletion in the values for Hb, Hct, RBC, MCV, MCH, and MCHC; while the WBC and platelet count were significantly (*p* < 0.05) increased in comparison with the negative control group. However, administration of the fenugreek (*T. foenum-graecum*) extract induced dose-dependent protective effects in the common carp by elevating the levels of Hb, Hct, RBC, MCV, MCH, and MCHC while reducing the values for WBC and platelet count at levels comparable with those for fish from the non-treated group.

### 3.6. Histological Observations

#### 3.6.1. Gills

**Figure 2:**
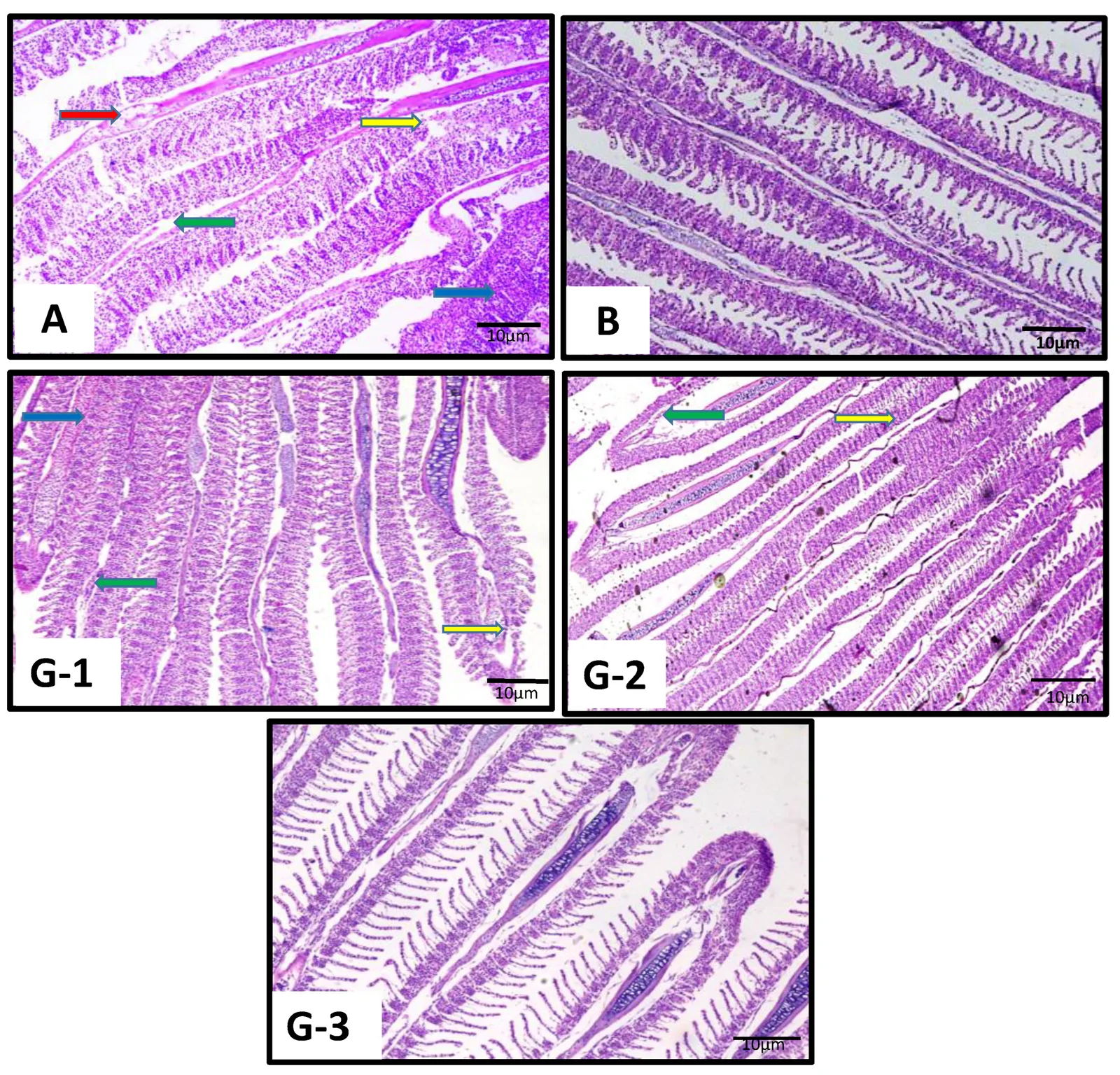
Photomicrographs (400×) of hematoxylin and eosin (H&E)-stained gill sections of the common carp (*C. carpio*) experimentally exposed for 28 days to CuO NPs (1.50 mg/L) as positive control (**A**; C-positive group), to no treatments as negative control (**B**; C-negative group), and to different concentrations of the fenugreek (*T. foenum-graecum*) extract at 100 mg/L (**G-1**), 125 mg/L (**G-2**), and 150 mg/L (**G-3**). Fish gills from the positive control showed various histological abnormalities including degenerative epithelium (green arrow), complete degeneration (red arrow), fused lamellae (blue arrow), and necrosis of primary lamella (yellow arrow). Fish from groups treated with the fenugreek showed dose-dependent curative effects on the gill epithelium. Scale bar = 10 µm. Histological observations were based on five randomly selected fields from one section per sample.

The gills of common carp from the positive control group exposed to CuO NPs showed severe histological abnormalities such as degenerative secondary lamellae (DSL), degenerative epithelium (DE), fused lamellae (FL), necrotic lamella (NL), necrosis of primary lamella (NPL), complete degeneration (CD), and complete lamellar fusion (CLF). However, the groups treated with different concentrations of *T. foenum-graecum* extract benefitted from ameliorative effects as revealed by the reduced histological abnormalities in the fish branchial epithelium in a dose-dependent manner (Figure 2). The intensity of various histological abnormalities in the fish gills is shown in Table 4.

#### 3.6.2. Liver

**Figure 3:**
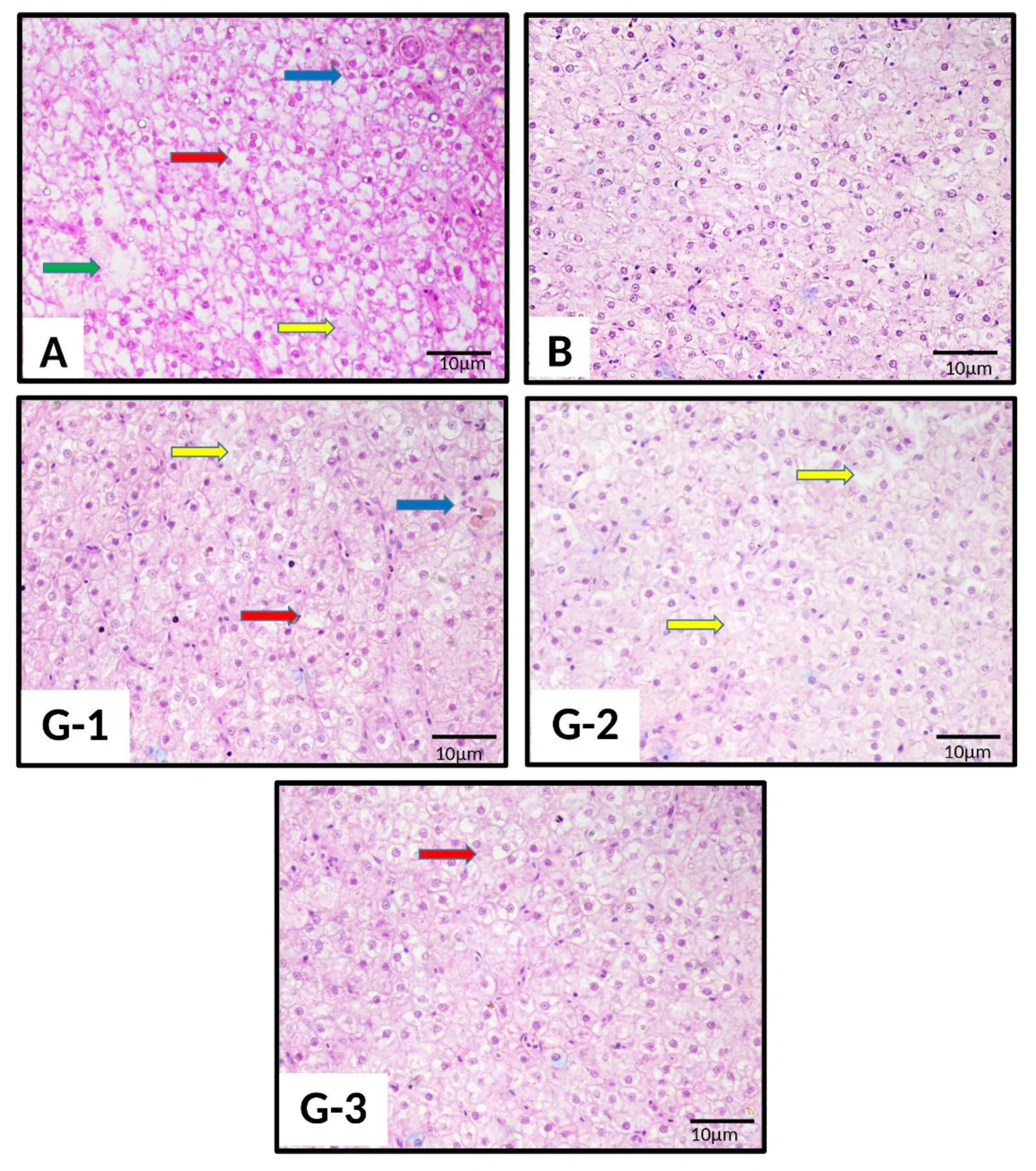
Photomicrographs (400×) of hematoxylin and eosin (H&E)-stained liver sections of the common carp (*C. carpio*) experimentally exposed for 28 days to CuO NPs (1.50 mg/L) as positive control (**A**; C-positive group), to no treatments as negative control (**B**; C-negative group), and to different concentrations of the fenugreek (*T. foenum-graecum*) extract at 100 mg/L (**G-1**), 125 mg/L (**G-2**), and 150 mg/L (**G-3**). Fish livers from the positive control showed various histological aberrations including vacuolization (red arrow); note that vacuolization identified by H&E staining alone cannot be definitively distinguished from physiological lipid or glycogen deposits, degenerative hepatocytes (yellow arrow), dilated sinusoid (blue arrow), and complete degeneration (green arrow). Fish from groups treated with the fenugreek showed dose-dependent curative effects in their livers. Scale bar = 10 µm. Histological observations were based on five randomly selected fields from one section per sample.

The livers of common carp from the positive control group exposed to CuO NPs exhibited various histological aberrations including degenerative hepatocytes (DH), vacuolization (V), damaged central vein (DCV), dilated sinusoid (DS), vacuolated degeneration (VD), and complete degeneration (CD). However, treatment with different doses of the *T. foenum-graecum* extract induced ameliorative effects in the fish by lowering the severity of histological abnormalities in the livers of the common carp (Figure 3). The intensities of different liver histological abnormalities are shown in Table 5.

#### 3.6.3. Kidney

**Figure 4:**
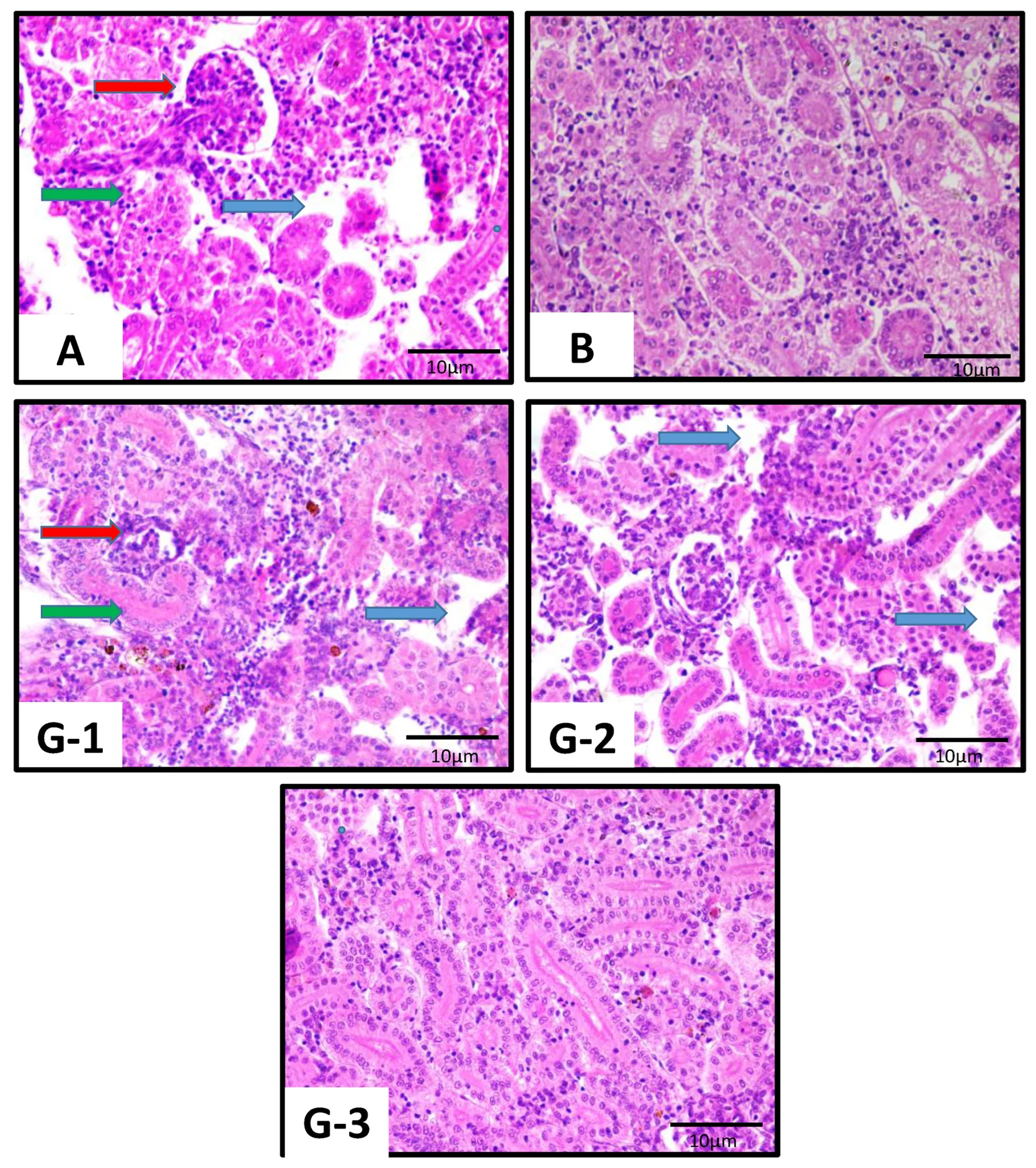
Photomicrographs (400×) of hematoxylin and eosin (H&E)-stained kidney sections of the common carp (*C. carpio*) experimentally exposed for 28 days to CuO NPs (1.50 mg/L) as positive control (**A**; C-positive group), to no treatments as negative control (**B**; C-negative group), and to different concentrations of the fenugreek (*T. foenum-graecum*) extract at 100 mg/L (**G-1**), 125 mg/L (**G-2**), and 150 mg/L (**G-3**). Fish kidneys from the positive control showed various histological abnormalities including swollen tubules (green arrow), abnormal glomerulus (red arrow), and complete degeneration (blue arrow). Fish from groups treated with the fenugreek showed dose-dependent curative effects in their kidneys even if some tissue damages were observed. Scale bar = 10 µm. Histological observations were based on five randomly selected fields from one section per sample.

The kidneys of common carp from the positive control group exposed to CuO NPs showed various histological aberrations including necrosis and tubular degeneration (NTD), abnormal glomerulus (AG), swollen tubules (ST), and complete degeneration (CD). However, exposure to three different doses of *T. foenum-graecum* extract induced curative effects in the fish by reducing the damages and enhancing the histological structure of their kidneys (Figure 4). The intensities of different histological abnormalities of kidneys are shown in Table 6.

### 3.7. Oxidative Stress Enzymes

In regard to the oxidative stress biomarkers, we found that the levels of LPO and GSH measured both in the liver and gills were significantly (*p* < 0.05) increased in the common carp exposed to CuO NPs with respect to fish from the negative control group, whereas a significant (*p* < 0.05) reduction in the CAT level was also observed. However, the treatment of fish with the *T. foenum-graecum* extract induced dose-dependent ameliorative effects in the oxidative stress responses by reducing both the LPO and GSH levels and enhancing the value for CAT at levels comparable to those recorded in *C. carpio* from the negative control group (Table 7).

## 4. Discussion

Nowadays, increasing concerns are being raised worldwide regarding the potential ecotoxicity of NPs to humans and the environment, mainly due to their deleterious impact on non-target organisms including fish [14,15,16]. Exposure to sub-lethal concentrations of metal NPs such as Ag NPs, CuO NPs, and Cd NPs provokes their bioaccumulation and biomagnification into aquatic food chains in addition to their toxicity, thus additionally posing a serious threat in terms of environmental sustainability [1]. Therefore, in addition to the increasing attention devoted to unraveling the hazards associated with NPs on aquatic systems, there is also an urgent need to discover novel therapeutic agents against NPs’ toxicity that remains a challenge. From this perspective, the present study was designed to assess the toxic impact of CuO NPs in the common carp (*C. carpio*) in terms of the hematological profile, antioxidant defense systems, and histology of fish tissues, and mostly with the aim to elucidate the ameliorative potential of fenugreek (*T. foenum-graecum*) seed extracts on the general health status of fish.

Plants produce a wide diversity of secondary metabolites that serve as defense or signal compounds and in a broad spectrum of other bioactivities. Due to the different biological and pharmacological properties of their secondary metabolites, some plants are referred to as medicinal plants and their extracts are commonly and efficiently used to treat infections, health disorders, or diseases [62]. According to the World Health Organization (WHO), about 70% of the world’s population depends on plants for their basic health care. As a matter of fact, in developing countries, herbal medicine is preferred to traditional medical methodologies in the treatment of diseases [63]. Moreover, many medicinal plants are known to be feasible sources of natural antioxidants, which primarily depend on numerous biologically active compounds that work collaboratively [64]. The results herein that we reported for the proximate composition of the seed extracts of *T.* *foenum-graecum* revealed that, as observed for other legumes, the fenugreek seeds were good sources of fats, proteins, and crude fibers. In detail, in *T.* *foenum-graecum* high percentages of carbohydrates and proteins were found together with a variety of phytochemicals, all of which were detected in significant amounts. These data were in line with those previously reported in the literature for fenugreek extracts [65,66] even though, conversely to Chauhan et al. [65], herein the presence of tannins was documented in *T.* *foenum-graecum*. The current findings were also in agreement with the work of Mbarki et al. [67], who confirmed the presence of flavonoids, phenols, and polysaccharides in fenugreek extracts, as well as with the results of the phytochemical study conducted by Benziane et al. [68], who reported the presence of tannins, saponins, flavonoids, and terpenes but a low presence of nitrogen compounds. Another similar work was carried out by Hwa et al. [69], who confirmed the presence of various phytochemicals and proximate components in the seeds of *T.* *foenum-graecum* including tannins, saponins, phenolics, and carbohydrates; however, they also reported the absence of flavonoids and reducing sugars.

In aquatic toxicology, the assessment of the biosafety of toxicants has vital importance. Trevan introduced the LD50 test (also known as the median lethal dose) in 1927, and it is nowadays the first test performed in every toxicity experiment because it determines the dose of a particular substance or chemical that caused the mortality of 50% of test animals within a specific period of time [70]. Therefore, this test is helpful in determining a relationship among concentrations of the administered substance and the mortality of test animals [71]. If a substance is more toxic, then its LD50 value would be lowered. Hence, a smaller dose of this substance would be required to cause mortality [72]. In the current study, the 96 h LC50 value of the *T.* *foenum-graecum* for *C. carpio*, namely 1226.98 ± 259.89 mg/L, indicated that the fenugreek seed extracts were toxic to the common carp at the higher concentrations examined herein. It is well known that death occurs when an animal model species is exposed to a substance at dosages exceeding its LC50 value [71]. For instance, Kumar et al. [73] inferred that the LC50 values of *Euphorbia* at 24, 48, 72, and 96 h for the fish *Heteropneustes fossilis* were 3.450 µL/L, 2.516 µL/L, 1.623 µL/L, and 1.315 µL/L, respectively. As a matter of fact, different plant species exhibit different LC50 values that are dependent on the secondary metabolites present in the plant as well as on the sensitive responses elicited by fish [74]. For common carp, Syngai et al. [75] examined the LC50 value of *Allium sativum* and found that it was 253.19 mg/L for *C. carpio*.

As previously mentioned, fenugreek (*T. foenum-graecum*) is a well-known traditional plant with medicinal properties and seeds that exhibit a powerful antioxidant activity both in vivo and in vitro [76,77] against oxidative stress induced by various toxins [36,37,78]. The findings of this study revealed that the experimental exposure to CuO NPs significantly reduced the body weight and length of the treated common carp, whereas the combined administration of the *T. foenum-graecum* extract mitigated the impact of the CuO NPs by triggering different protective effects. Interestingly, it was noted that all the tested doses of fenugreek induced a significant augmentation of the total fish weight and length, especially the intermediate administrated dosage of 125 mg/L. The results herein reported were also supported by Alloui et al. [79], who used *T. foenum-graecum* extract as a growth promoter in broiler chicken and reported their increase in growth rate after the administration of the fenugreek extract. Similarly, it was documented that dietary administration of fenugreek seeds promoted the growth performance of gilthead seabreams [33]. Therefore, these data made it possible for us to hypothesize that fenugreek plays an important role in optimizing the absorption and use of food content, which may result in increased production if regularly adopted in fish aquaculture.

Moreover, it is broadly known that evaluation of the hematological profile is a valuable tool for fishery biologists to assess fish health and monitor potential stress responses [50]. In this study, it was noteworthy that CuO NPs also provoked toxic effects on the hematological parameters of the exposed fish while the treatment with various doses of the fenugreek extract induced dose-dependent curative and protective effects in the common carp, elevating the levels of Hb, Hct, RBC, MCV, MCH, and MCHC, as well as reducing the values for WBC and platelet count to levels comparable with those of fish from the non-treated group. Of relevance, gilthead seabream specimens fed with different doses of a dietary supplement of fenugreek showed an increment in RBC counts [33]. Therefore, it is feasible to hypothesize that these augmentations in blood parameters were a result of the effective role that fenugreek played as a natural antioxidant [80]. Similar findings were also documented by Abdel-Daim et al. [32], who demonstrated the protective activities of *T. foenum-graecum* in rats exposed to deltamethrin by modulating the hematological, biochemical, and oxidative stress parameters. The current results were also in good agreement with the work of Gupta et al. [81], who revealed that fenugreek protected against selenite-induced cataracts by virtue of its antioxidant properties. Likewise, the present results were also in line with the findings of Elseed et al. [82], who reported that inclusion in the diet of a *T. foenum-graecum* saponin extract had positive effects on the production, hematological parameters, and blood metabolites of rabbits.

Furthermore, based on the histological observations, we also found a recovery tendency from CuO-NP-induced tissue damage in the gills, livers, and kidneys of common carp in a dose-dependent manner after treatment with the fenugreek extract. The liver is very important organ that performs vital functions including detoxification, synthesis of numerous components of blood plasma, storage of glycogen, and release of glucose to the blood [83,84,85,86]; gills are crucial for respiratory gas exchange activity, osmoregulation, active ion transport, acid–base balance and excretion of nitrogenous wastes [84,85]; whereas kidneys are the primary excretory and osmoregulatory organ in fish [87]. Therefore, the maintenance of the histological stability and integrity of these organs is critical for proper nutrition, defense, and fish growth. Similar histological results were also reported in the work of Waffa et al. [34], who found that *T. foenum-graecum* induced protective effects in Nile tilapia after exposure to cadmium by reducing the morphological damages observed in fish gills, livers, and muscles. As further support of these data, relevant hepato- and nephro-protective effects of the fenugreek seed extract were also reported in rats against sodium nitrate toxicity [88]. Moreover, the histological results herein reported were also in agreement with the findings of Belaïd-Nouria et al. [37], who also demonstrated the hepato-protective effects of fenugreek seeds against aluminum chloride toxicity in rats.

The authors also acknowledge that vacuolization observed in liver sections (Figure 3) was interpreted based solely on hematoxylin and eosin (H&E) staining. Although the vacuolated hepatocytes exhibited cytoplasmic disintegration, nuclear pyknosis, and loss of normal cord arrangement, supporting their interpretation as degenerated cells rather than physiological lipid or glycogen deposits, it is recognized that H&E staining alone cannot definitively distinguish between these entities. This methodological limitation is noted, and all interpretations of vacuolization in this study remain cautious and evidence-based.

Moreover, in the present study, the exposure to CuO NPs in the common carp significantly altered the levels of CAT, LPO, and GSH enzymes in response to the oxidative stress condition induced by CuO NP exposure. Noteworthy, the combined treatment of CuO NPs with different concentrations of *T. foenum-graecum* extract induced ameliorative antioxidant effects in the fish as demonstrated by the level of oxidative stress enzymes that were found at values comparable to those recorded in common carp from the non-treated group. This result may be explained by the presence of antioxidant molecules in the fenugreek seeds, which play a crucial role in the maintenance of cellular antioxidant status [89]. The effectiveness of dietary supplements of fenugreek to enhance the oxidative defense response in fish in a dose-dependent manner was previously reported in gilthead seabreams, showing an increase in the expression of the main antioxidative enzyme genes with an increase in the dosage of fenugreek in the diets [33]. Since there is now a growing interest in the role and usage of antioxidants from natural products as a strategy to prevent oxidative damage in a variety of health disorders, such antioxidant properties of fenugreek are gaining more attention worldwide and have been linked to a variety of health benefits [89].

Overall, the beneficial effects observed in the common carp reared with different doses of fenugreek extracts, especially at the concentration of 125 mg/L, in mitigating the toxicity of CuO NPs in most of the parameters evaluated herein may likely have been related to the dose-dependent antioxidant and protective potential of *T. foenum-graecum,* which enhances free radical scavenger enzymatic activities and reduces the production of ROS [29,30,31,32]. This high antioxidant property of fenugreek may be due to its characteristic polyphenolic profile, which serves in various functions including as an antioxidant, scavenger of free radicals, chelating agent, and modifier of numerous enzymatic and biological reactions [90].

## 5. Conclusions

The present study illustrated the protective role of a fenugreek (*T. foenum-graecum*) extract against the toxicity of CuO NPs in the common carp (*C. carpio*). In detail, it was revealed that the *T. foenum-graecum* extract induced ameliorative effects in the general health status of fish by modulating the hematological parameters and oxidative stress enzymes and by decreasing histopathological lesions in the structures of fish gills, livers, and kidneys after reducing the accumulation of Cu. In the present work, the fenugreek (*T. foenum-graecum*) extract was administrated in a dose-dependent manner in order to evaluate its use as a novel therapeutic agent due to the fact that the practice of aquaculture is intensively increasing worldwide along with the spreading of infectious diseases. Overall, the findings in this work confirmed the ameliorative and protective effects of the fenugreek (*T. foenum-graecum*) extract against the toxicity of NPs in aquatic organisms. Therefore, all the beneficial physiological properties exerted by fenugreek as a dietary supplement provide evidence of its promising nutraceutical value and applicative potential, especially in the sector of fish aquaculture. Indeed, fenugreek can improve the growth performance and wellness of organisms with a further advantage of favoring environmentally friendly organic production.

## Figures and Tables

**Table 1:** Effects of CuO NPs and different concentrations of the fenugreek (*T. foenum-graecum*) extract on the body weight and length of the common carp (*C*. *carpio*) experimentally exposed for 28 days. Data are expressed as means ± standard error (SE; n = 10).

Groups	Concentration of CuO NPs (mg/L)	Concentration of Fenugreek Extract (mg/L)	Initial Weight (g)	Final Weight (g)	Initial Length (cm)	Final Length (cm)
C-positive	1.50	0.00	45.28 ± 0.34	43.75 ± 0.41 ^c^	14.40 ± 0.56	13.57 ± 0.67 ^c^
G-1	1.50	100.00	45.96 ± 0.21	47.09 ± 0.67 ^a^	14.90 ± 0.30	15.97 ± 0.49 ^a^
G-2	1.50	125.00	46.25 ± 0.90	49.03 ± 0.50 ^a^	15.30 ± 0.95	18.10 ± 0.62 ^a^
G-3	1.50	150.00	45.43 ± 0.62	47.98 ± 0.43 ^b^	14.17 ± 1.05	17.00 ± 0.36 ^b^
C-negative	0.00	0.00	44.99 ± 0.24	49.33 ± 0.56 ^a^	14.38 ± 0.55	18.99 ± 0.66 ^a^

Means with different letters (a, b, c) in the same column differ significantly (*p* < 0.05).

**Table 2:** Concentration of Cu (µg/kg ww) in gills, livers, and kidneys of the common carp (*C. carpio*) experimentally exposed to CuO NPs and different concentrations of the fenugreek (*T. foenum-graecum*) extract for 28 days. Data are expressed as means ± standard error (SE; n = 10).

Groups	Concentration of CuO NPs (mg/L)	Concentration of the Fenugreek Extract (mg/L)	Level of Cu (µg/kg ww) in Gills	Level of Cu (µg/kg ww) in Liver	Level of Cu (µg/kg ww) in Kidney
C-positive	1.50	0.00	1.18 ± 0.006	1.38 ± 0.006	0.05 ± 0.006
G-1	1.50	100.00	1.17 ± 0.010	1.36 ± 0.010	0.04 ± 0.010
G-2	1.50	125.00	1.12 ± 0.010	1.28 ± 0.010	0.03 ± 0.010
G-3	1.50	150.00	1.09 ± 0.010	1.19 ± 0.010	0.03 ± 0.010
C-negative	0.00	0.00	0.12 ± 0.01	0.09 ± 0.02	0.11 ± 0.04

**Table 3:** Values of different hematological parameters of the common carp (*C. carpio*) experimentally exposed to CuO NPs and different concentrations of the fenugreek (*T. foenum-graecum*) extract for 28 days. Data are expressed as means (SE; n = 10).

Groups	Concentration of CuO NPs (mg/L)	Concentration of Fenugreek Extract (mg/L)	Hb (g/dL)	Hct (%)	RBC (×10^6^/M)	WBC (×10^3^/µL)	MCV (fl)	MCH (pg)	MCHC (g/dL)	Platelet Count (×10^3^/µL)
C-positive	1.50	0.00	5.6	18.16	1.083	68.987	129.4	34.03	26.96	1272.3
G-1	1.50	100.00	7.067	22.73	1.807	3.497	140.4	40.12	33.57	209.7
G-2	1.50	125.00	7	22.5	1.823	2.57	145.9	40.35	34.27	207.5
G-3	1.50	150.00	6.967	23.73	1.79	1.497	146.4	41.96	34.34	206.4
C-negative	0.00	0.00	9.21	24.62	2.82	3.34	139.41	42.31	34.11	181.20

**Table 4:** Comparison of various histological abnormalities in the gills of common carp from control and treated groups. Please note that five fields of one histological section per sample were analyzed.

Histological Alterations in Gills	Control	C-Positive	G-1	G-2	G-3
Degenerative secondary lamellae (DSL)	-	++	+	-	-
Degenerative epithelium (DE)	-	+++	++	+	+
Fused lamellae (FL)	-	+++	++	+	+
Necrotic lamella (NL)	-	+++	+	+	-
Necrosis of primary lamella (NPL)	-	++	-	-	-
Complete degeneration (CD)	-	+++	+	-	-
Complete lamellar fusion (CLF)	-	++	+	-	-

No symptoms (-); very severe (+++); less severe (++); mild (+).

**Table 5:** Comparison of various histological abnormalities in the livers of common carp from control and treated groups. Please note that five fields of one histological section per sample were analyzed.

Histological Alterations in Liver	Control	C-Positive	G-1	G-2	G-3
Hepatocytes (DH)	-	++	+	+	+
Vacuolization (V)	-	+++	+	-	+
Damaged central vein (DCV)	-	+++	++	+	-
Dilated sinusoid (DS)	-	+++	+	-	-
Vacuolated degeneration (VD)	-	++	-	-	-
Complete degeneration (CD)	-	+++	-	-	-

No symptoms (-); very severe (+++); less severe (++); Mild (+).

**Table 6:** Comparison of various histological abnormalities in the kidneys of common carp from control and treated groups. Please note that five fields of one histological section per sample were analyzed.

Histological Alterations in Kidney	Control	C-Positive	G-1	G-2	G-3
Necrosis and tubular degeneration (NTD)	-	++	+	+	+
Abnormal glomerulus (AG)	-	+++	-	+	-
Swollen tubules (ST)	-	+++	+	+	+
Complete degeneration (CD)	-	+++	+	-	-

No symptoms (-); very severe (+++); less severe (++); mild (+).

**Table 7:** Concentrations of different oxidative stress enzymes (lipid peroxidation (LPO) as nmol/mg protein of MDA; glutathione (GSH) as µM/g of tissue homogenate; catalase (CAT) as unit per mg (U/mL) of tissue homogenate) in livers and gills of the common carp (*C. carpio*) experimentally exposed to CuO NPs and different concentrations of the fenugreek (*T. foenum-graecum*) extract for 28 days. Data are expressed as means ± standard error (SE; n = 10).

Groups	Concentration of CuO NPs (mg/L)	Concentration of Fenugreek Extract (mg/L)	LPO		GSH		CAT	
			Liver	Gills	Liver	Gills	Liver	Gills
C-positive	1.50	0.00	545.80 ± 20.09	759.4 ± 22.05	2040 ± 31.04	8007 ± 56.11	2.11 ± 0.21	1.43 ± 0.05
G-1	1.50	100.00	382.40 ± 17.03	337.3 ± 15.04	1340 ± 23.06	2246 ± 35.82	2.47 ± 0.34	2.78 ± 0.12
G-2	1.50	125.00	364.90 ± 15.04	350 ± 15.08	1343 ± 23.09	2269 ± 35.32	2.39 ± 0.26	2.62 ± 0.16
G-3	1.50	150.00	385.40 ± 16.01	366.6 ± 16.04	1455 ± 25.01	2294 ± 36.12	2.34 ± 0.22	2.46 ± 0.11
C-negative	0.00	0.00	319.11 ± 5.11	423.1 ± 3.14	1235 ± 8.15	2239 ± 8.55	2.37 ± 0.02	2.54 ± 0.01

## Data Availability

The data presented in this study are available upon request from the corresponding author.
